# Structural insights into the RNA methyltransferase domain of METTL16

**DOI:** 10.1038/s41598-018-23608-8

**Published:** 2018-03-28

**Authors:** Agnieszka Ruszkowska, Milosz Ruszkowski, Zbigniew Dauter, Jessica A. Brown

**Affiliations:** 1 Department of Chemistry and Biochemistry, University of Notre Dame, Notre Dame, IN 46556 USA; 2 Synchrotron Radiation Research Section of MCL, National Cancer Institute, Argonne, IL 60439 USA

## Abstract

*N*^6^-methyladenosine (m^6^A) is an abundant modification in messenger RNA and noncoding RNAs that affects RNA metabolism. Methyltransferase-like protein 16 (METTL16) is a recently confirmed m^6^A RNA methyltransferase that methylates U6 spliceosomal RNA and interacts with the 3′-terminal RNA triple helix of MALAT1 (metastasis-associated lung adenocarcinoma transcript 1). Here, we present two X-ray crystal structures of the N-terminal methyltransferase domain (residues 1–291) of human METTL16 (METTL16_291): an apo structure at 1.9 Å resolution and a post-catalytic *S*-adenosylhomocysteine-bound complex at 2.1 Å resolution. The structures revealed a highly conserved Rossmann fold that is characteristic of Class I *S*-adenosylmethionine-dependent methyltransferases and a large, positively charged groove. This groove likely represents the RNA-binding site and it includes structural elements unique to METTL16. In-depth analysis of the active site led to a model of the methyl transfer reaction catalyzed by METTL16. In contrast to the major m^6^A methyltransferase heterodimer METTL3/METTL14, full-length METTL16 forms a homodimer and METTL16_291 exists as a monomer based on size-exclusion chromatography. A native gel-shift assay shows that METTL16 binds to the MALAT1 RNA triple helix, but monomeric METTL16_291 does not. Our results provide insights into the molecular structure of METTL16, which is distinct from METTL3/METTL14.

## Introduction

*N*^6^-methyladenosine (m^6^A) is the product of a dynamic and abundant modification in eukaryotic messenger RNA (mRNA) and noncoding RNAs (ncRNA)^1^ that affects RNA stability^2^, pre-mRNA processing^3^, microRNA biogenesis^4^ and translation efficiency^5^. Approximately three to five m^6^A sites occur in each mRNA molecule, predominantly within 3′-untranslated regions and near stop codons^6,7^. Most m^6^A sites lie within a highly conserved RRACH (R = A or G; H = A, C or U) sequence motif^1,8^ and are regulated by adenosine methyltransferases (‘writers’), m^6^A-binding proteins (‘readers’) and m^6^A-demethylating enzymes (‘erasers’)^9^.

Most m^6^A RNA marks are catalyzed by a heterodimeric ‘writer’ complex comprised of methyltransferase-like protein 3 and methyltransferase-like protein 14 (METTL3/METTL14), which specifically methylates adenosine within the RRACH motif with no obvious structural preferences^10^. Recently, methyltransferase-like protein 16 (METTL16) was confirmed to be an m^6^A RNA methyltransferase that modifies U6 spliceosomal RNA^11,12^ and the MAT2A mRNA encoding *S*-adenosylmethionine (SAM) synthase^11,13^. In addition, METTL16 also binds to ribosomal RNA, mRNA, and long ncRNAs, such as X-inactive specific transcript and the 3′ triple helix of metastasis-associated lung adenocarcinoma transcript 1 (MALAT1)^12,14^. Unlike METTL3/METTL14, METTL16-dependent m^6^A marks do not occur within the RRACH sequence motif and they are found in introns and at intron-exon boundaries^11,12^. Preliminary studies suggest that METTL16 uses a combination of sequence and structure to recognize its RNA substrates^11^. Interestingly, one study has proposed that METTL16 has evolved an additional role in pre-mRNA splicing, allowing METTL16 to function as both an m^6^A ‘writer’ and ‘reader’^11^. As an m^6^A ‘writer’, METTL16 rapidly methylates the MAT2A mRNA in the presence of SAM, leading to intron retention followed by nuclear degradation^11^. When SAM levels are low, prolonged occupation of METTL16 on MAT2A mRNA enhances splicing of retained intron^11^.

METTL16 homologs are found from *Escherichia coli* to human and they all possess an N-terminal methyltransferase domain^11,15–17^. As a SAM-dependent methyltransferase (SAM-MTase), METTL16 is predicted to have a highly conserved Rossmann fold^18^. In characterized MTases, the conserved Rossmann fold is composed of alternating β strands and α helices, forming a seven-stranded β sheet that is sandwiched between clusters of α helices. In general, the SAM-binding site is usually found in the N-terminal segment of the β sheet, whereas the substrate-binding site lies within the C-terminal segment^19^. Although MTases are structurally similar, they use a variety of mechanisms to specifically recognize their targets, such as oligomerization and unique structural elements. These unique structural elements include variable loop regions within the Rossmann fold and auxiliary domains within and/or flanking the Rossmann fold^20–22^.

In this work, we present the structural analysis of human METTL16 based on two X-ray crystal structures of the N-terminal MTase domain (residues 1–291) of human METTL16 (METTL16_291): an apo form at 1.9 Å resolution and a post-catalytic complex with *S*-adenosylhomocysteine (SAH) at 2.1 Å resolution. We analyzed the salient MTase features of METTL16_291 based on characterized SAM-MTases; this analysis highlights the unique attributes of METTL16_291 and contrasts it to the structural studies of METTL3/METTL14. Moreover, we investigated the oligomeric state of METTL16 and its ability to bind to the MALAT1 RNA triple helix.

## Results and Discussion

### METTL16_291 is a SAM-dependent MTase with a conserved Rossmann fold

**Figure 1:**
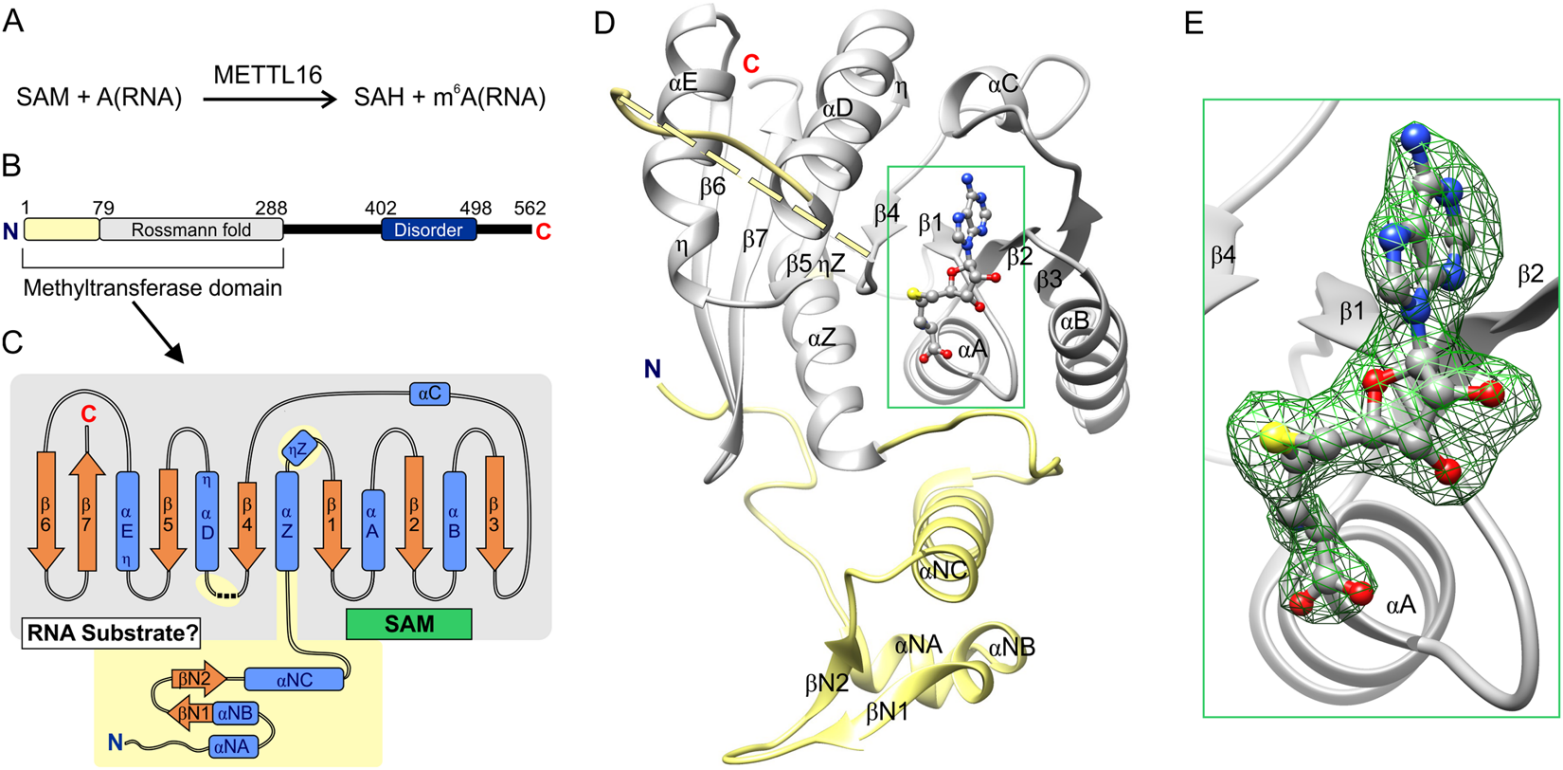
Structural overview of METTL16_291. (**A**) General scheme of methyl transfer catalyzed by METTL16. (**B**) Schematic diagram of the overall domain organization of human METTL16 shows the unique N-terminal region (residues 1–78, yellow) and the conserved Rossmann fold (residues 79–288, gray) in the methyltransferase domain, and a region (residues 402–498, dark blue) predicted to be disordered by the MobiDB server^41^. Blue N and red C indicate termini. (**C**) Schematic diagram of the secondary structure of the methyltransferase domain from METTL16_291. Blue boxes represent α and 3_10_ (η) helices, orange arrows represent β strands and dashed lines represent residues 188–214 that cannot be modeled. Binding sites for SAM (or SAH) and RNA are labeled within Rossmann fold. (**D**) Overall fold of SAH-bound METTL16_291 revealed nine β strands, nine α helices (two containing 3_10_ twist at end of helix) and one 3_10_ helix. The conserved Rossmann fold of SAM-MTases is colored in gray; the structural elements colored yellow are unique to METTL16. (**E**) Close-up view of SAH (gray ball-and-stick representation); green mesh represents OMIT *F*_*o*_*-F*_*c*_ electron density map of SAH contoured at 4 σ level.

In general, m^6^A RNA MTases bind to SAM and RNA, transfer a methyl group from SAM to adenosine, and then the methylated RNA and SAH products dissociate from the MTase (Fig. 1A). To provide structural insights into the methyltransferase reaction catalyzed by the MTase domain of METTL16, we solved X-ray crystal structures of apo and SAH-bound METTL16_291 (METTL16_291/SAH, Fig. 1 and Table 1). These structures confirmed that METTL16 is a Class I SAM-MTase that uses a conserved Rossmann fold to bind the SAH product and likely SAM, the methyl-donor substrate (Figs. 1B–E and S1). The conserved secondary structural elements of METTL16_291 are numbered according to the canonical SAM-MTase fold^19^ (Fig. 1C,D). The conserved SAM-MTase core (residues 79–288) of METTL16_291 possesses a mostly parallel, seven-stranded β sheet (β1–β7) organized as ‘3214576’, with β7 being antiparallel (Fig. 1B–D). The β sheet appears to be stabilized by a disulfide bridge between C183 and C247, thereby linking the β4 and β5 strands. This disulfide bridge is present at approximately 40% occupancy in the apo structure and is absent in the SAH-bound complex, likely because of radiation damage and/or the presence of Tris(2-carboxyethyl)phosphine hydrochloride (TCEP**)** in the protein buffer. Including TCEP was necessary to prevent protein precipitation. The ‘3214576’ β sheet is packed between two clusters of α and 3_10_ (η) helices (Fig. 1C,D). Although the MTase domain of METTL16 has a canonical Class I MTase structure, METTL16_291 has several unique structural regions: residues 1–78 that precede the Rossmann fold as well as residues 95–97 (ηZ) and 188–222 (a putative loop containing disordered residues 188–214) within the Rossmann fold (Fig. 1C,D). Altogether, these structural elements unique to METTL16 likely contribute to its RNA substrate specificity that is distinctly different from the METTL3/METTL14 complex^6,7,10–12,22–24^.

**Table 1:** Data collection and refinement statistics.

Human METTL16 (1–291)	Apo	+SAH
**Data collection**		
Wavelength (Å)	1.0000	1.0000
Space group	*I*4_1_32	*I*4_1_32
Unit cell parameters		
*a* = *b* = *c* (Å)	190.1	190.4
Resolution (Å)	50–1.94 (2.05–1.94)	50–2.10 (2.23–2.10)
Unique reflections	43441 (6791)	34224 (5267)
Multiplicity	16.2 (14.2)	17.3 (14.2)
Completeness (%)	99.7 (98.2)	99.3 (95.9)
*R*_meas_^a^(%)	6.9 (141.8)	6.6 (141.5)
<*I*/σ(*I)*>	25.0 (2.0)	28.7 (2.0)
**Refinement**		
*R*_free_ reflections	1000	1027
*R*_work_/*R*_free_ (%)	18.2/21.0	18.2/21.9
No. of atoms (non-H)	2414	2386
protein	2194	2163
ligands	13	42
solvent	207	181
Average B-factor (Å^2^)	48.7	54.0
RMSD from ideal geometry		
bond lengths (Å)	0.014	0.014
bond angles (°)	1.6	1.7
Ramachandran statistics (%)		
favored	96.6	96.2
allowed	3.4	3.8
outliers	0.0	0.0
PDB ID	6b91	6b92

^a^*R*_meas _ = redundancy independent R-factor^70^.

Values in parentheses correspond to the highest resolution shell.

### Cofactor-binding site is defined by extensive network of noncovalent interactions

As an m^6^A RNA MTase, METTL16 interacts with three different ligands: SAM, SAH and RNA (Fig. 1A)^11,12^. To study ligand binding, we soaked METTL16_291 crystals with SAM, SAH, adenosine 5′-monophosphate (a mimic of the methyl-acceptor adenosine) and various RNAs but obtained only a SAH-bound complex after soaking crystals with SAM. The presence of SAH, rather than SAM, was determined based on calculations of electron density maps (Fig. 1E) using SAM and SAH each as models (see Methods).

**Figure 2:**
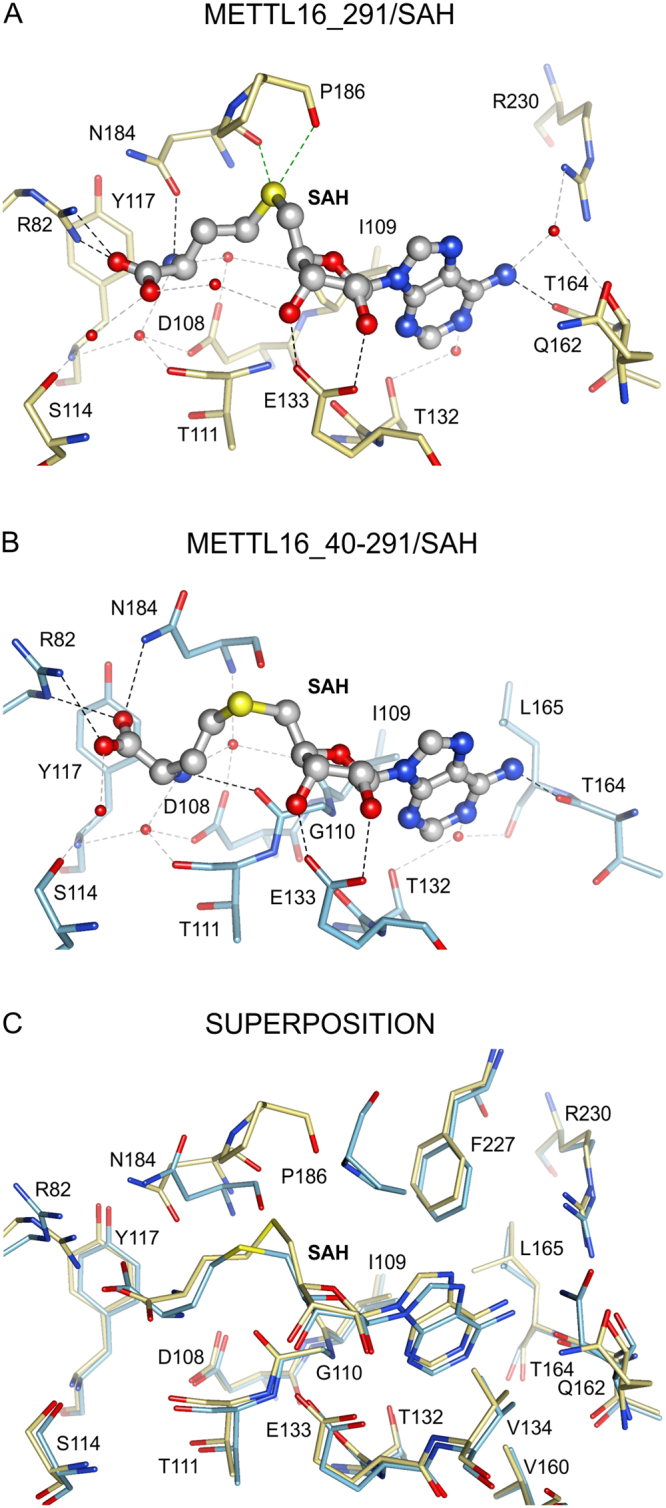
Interactions between METTL16_291 and SAH. The SAH-binding sites are shown for (**A**) METTL16_291/SAH complex (yellow sticks, PDB ID: 6b92), (**B**) METTL16_40–291/SAH (blue sticks, PDB ID: 2h00^27^) and (**C**) superposition of METTL16_291/SAH and METTL16_40–291/SAH. Views of SAH-binding pocket are presented in the same orientation in all panels. Black, gray and green dashed lines respectively represent noncovalent interactions via hydrogen bonds, water-mediated hydrogen bonds and hypervalent sulfur-oxygen bonds^25^ between SAH atoms and residues of METTL16. Water molecules are shown as red spheres. All residues forming the hydrophobic pocket (I109, V134, V160, L165 and F227) around the adenine moiety of SAH are shown together in panel C.

Our crystal structure shows that METTL16_291 binds SAH, and likely SAM as well, within a deep pocket of the Rossmann fold (Figs. 1D,E and 2A). Multiple hydrogen bonds ensure the proper positioning of the adenine, ribose and homocysteine moieties of SAH (Fig. 2A). The adenine moiety of SAH is recognized by the main chain of T164 in addition to a hydrophobic pocket created by the side chains of I109, V134, V160, L165 and F227 (Fig. 2). The 2′- and 3′-hydroxyl groups of the ribose are recognized via hydrogen bonding to E133, a universally conserved residue in SAM-MTases (Figs. 2A and S1)^19^. The sulfur atom of SAH likely hypervalently interacts^25^ with the backbone carbonyl oxygen atoms of N184 and P186, which are part of the conserved catalytic motif (see below). R82 forms a salt bridge with the carboxyl group of SAH. Moreover, six water molecules mediate hydrogen-bonding interactions between SAH and D108, I109, T111, S114, Y117, T132, Q162 and R230 (Fig. 2A). Lastly, the nucleotide-binding site includes the conserved *GXG* motif (G110-T111-G112) that is characteristic of the SAM-MTase core (Fig. S1)^19,26^. This motif shapes the cavity between adenosine and the homocysteine moieties but only T111 interacts with SAH via a water-mediated hydrogen bond (Fig. 2A).

Next, we compared the SAH-binding sites of our METTL16_291/SAH structure and an unpublished X-ray crystal structure of METTL16 containing residues 40–291 (METTL16_40–291/SAH, Fig. 2B, PDB ID: 2h00, Structural Genomics Consortium^27^). From this comparative analysis, we discovered that most METTL16-SAH interactions overlap (Fig. 2C). However, the homocysteine moiety in METTL16_40–291, particularly the location of the sulfur atom (shifted by 2.3 Å in comparison to METTL16_291), is reoriented due to the different conformations of R82 and N184 (Fig. 2C). Further analysis of SAH-bound METTL16_291 with X-ray crystal structures of other Class I SAM-MTases (PDB ID: 1boo^28^, 1qan^29^, 1eiz^30^, 1hnn^31^, and 1khh^32^) indicates that the sulfur atom location of SAH in our complex is unique, which may be a crystallization artifact resulting from an interaction between R82 and E217 of a symmetrically related molecule in the crystal lattice. Importantly, the conformation of SAH in METTL16_40–291/SAH, as preserved in other SAM-MTases, is properly oriented after a productive methyl transfer reaction. Thus, these structural snapshots suggest that METTL16_40–291/SAH represents a post-catalytic state that likely occurs before the state captured in METTL16_291/SAH.

Structural comparisons of apo and SAH-bound METTL16_291 structures revealed that the cofactor-binding site in the apo structure is occupied by four water molecules, which serve as placeholders for the following heteroatoms: (i) amino and carboxyl groups of the homocysteine moiety, (ii) 3′-hydroxyl of the ribose moiety and (iii) N1 of adenine. Altogether, our structures indicate that METTL16 uses an extensive network of noncovalent interactions to facilitate binding of SAH and likely SAM as well.

### METTL16 has a large, positively charged groove to interact with RNA

**Figure 3:**
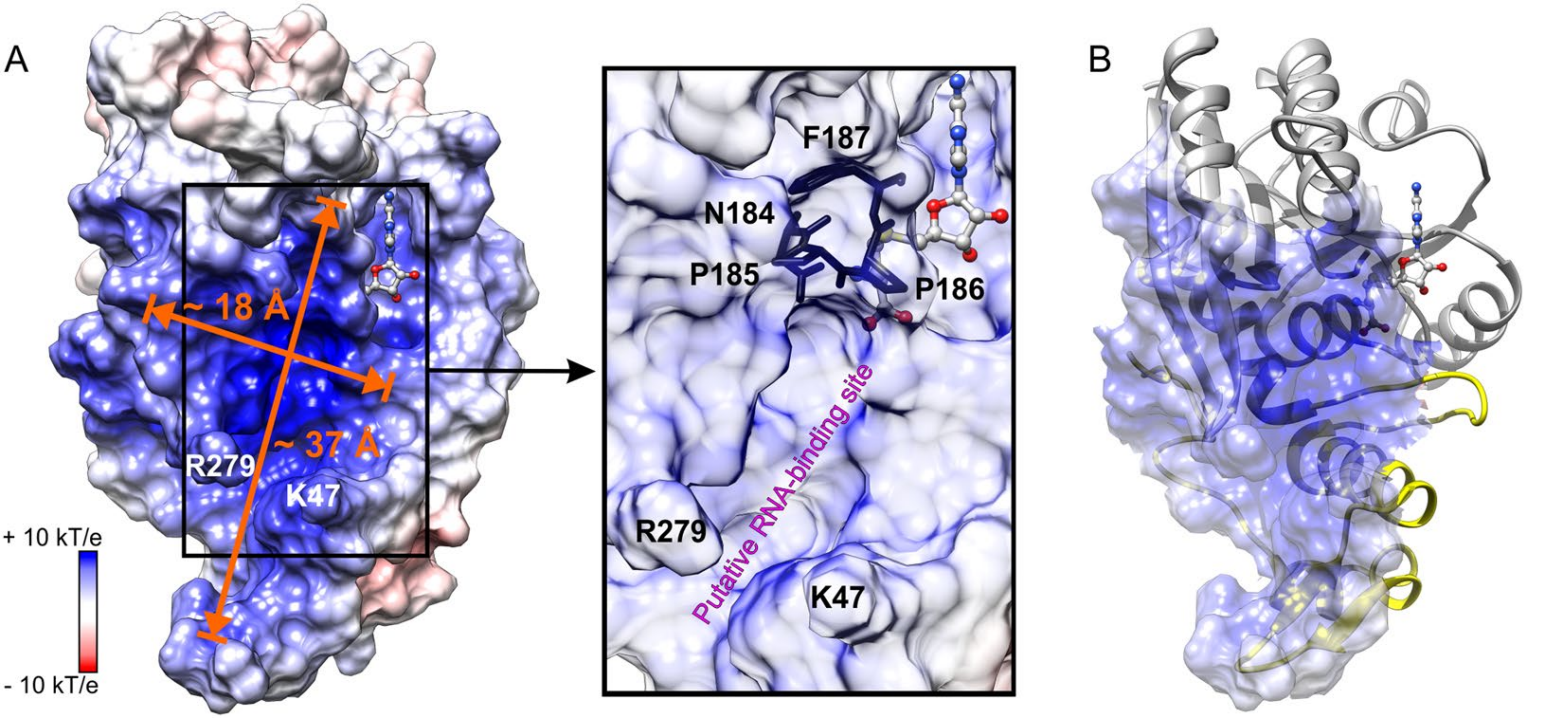
Putative RNA-binding site in METTL16_291. (**A**) Surface electrostatic potential is shown for the METTL16_291/SAH complex. Groove dimensions and claw-forming residues, K47 and R279, are marked and SAH is a ball-and-stick model. The box shows a close-up view of the conserved catalytic residues, _184_NPPF_187_, located between the putative RNA-binding site and SAH-binding pocket under semi-transparent surface. (**B**) Overall fold of METTL16_291 with semi-transparent representation of positively charged groove surface. Gray and yellow colors are as defined in Fig. 1.

To identify a putative RNA-binding site, we inspected the surface electrostatic potential of METTL16_291/SAH using the PDB2PQR server^33^ and found a positively charged groove covering an area of ~2000 Å^2^, which could interact favorably with the negatively charged phosphate backbone of RNA (Fig. 3A). Residues lining the groove are highly conserved among *Chordata* METTL16 homologs (Fig. S1), thereby supporting the putative functional importance of this groove. The positively charged surface of METTL16 includes K5, K14, R41 and K47 in the unique N-terminal region; R82, K251, K252, R279 and R282 in the conserved Rossmann fold; and dipoles of the helices αNA, αNC, αZ, αηD and ηαE (Figs. 1D and 3B). The N-terminal residues 1–78 most likely contribute to the unique RNA substrate specificity of METTL16. Interestingly, residues K47 and R279 protrude from the surface to form a claw-like structure wide enough (~8 Å) to accommodate a phosphate group in the RNA backbone (Fig. 3A).

With our current structures of METTL16, it is not possible to predict how METTL16 recognizes its RNA substrates for three reasons. First, METTL16 may undergo conformational changes upon RNA binding, as observed for the 5-methylcytosine DNA methyltransferase from *Haemophilus haemolyticus*^34^. Second, the C-terminal domain of METTL16 (residues 292–562) is missing in our structure and may also contribute to substrate recognition. Third, the RNA substrate specificity of METTL16 is not yet clear. The m^6^A sites in U6 snRNA and MAT2A mRNA, two confirmed substrates, reside in single-stranded bulges flanked by double-stranded RNA^35,36^. The active site of METTL16_291/SAH can easily accommodate single-stranded RNA within a ~37 × 18 Å groove (Fig. 3A). Moreover, the conserved residues of METTL16 essential for catalysis, _184_NPPF_187_, are located between the SAM/SAH-binding site and the positively charged groove, suggesting that this region is indeed the active site of METTL16 (Fig. 3A). Thus, the putative RNA-binding site of METTL16 is compatible with the canonical SAM-MTase architecture, whereby the RNA substrate-binding site is located within the C-terminal segment of the parallel β sheet and the SAM-binding site is situated within the N-terminal segment of the parallel β sheet (Fig. 1C)^19^.

### METTL16 uses a conserved mechanism to catalyze methyl transfer

**Figure 4:**
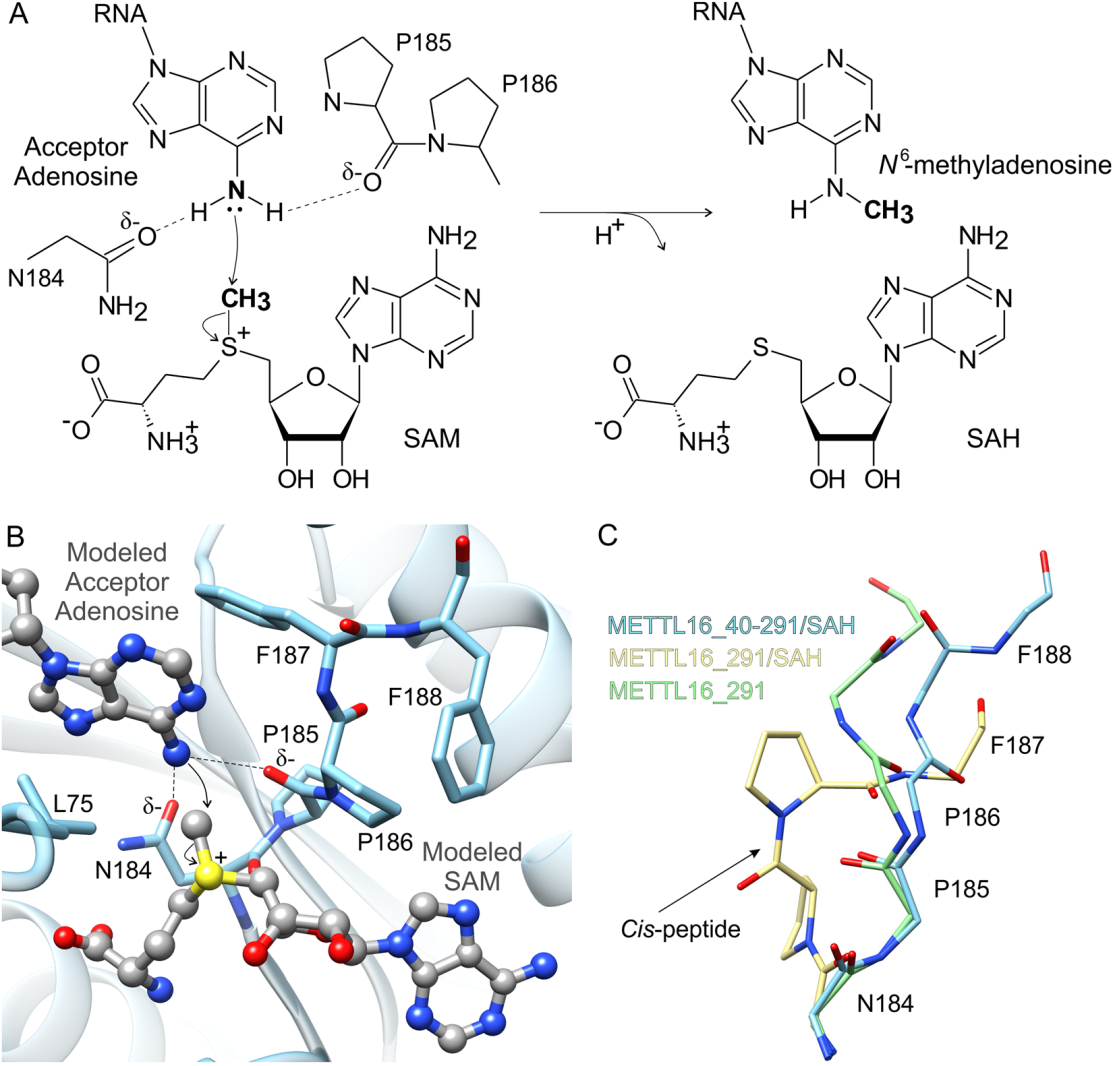
Proposed mechanism of methyl transfer catalyzed by METTL16. (**A**) A schematic of the chemical S_N_2 reaction of methyl transfer from SAM to *N*^6^-adenosine is shown with curved arrows to indicate movement of electron pairs. (**B**) To model methyl transfer, SAM (gray ball-and-stick representation) was modeled into the SAH-binding site of METTL16_40–291 (blue) and the methyl-acceptor adenosine (gray ball-and-stick representation) was modeled via superposition of an m^6^A DNA MTase, EcoP15l (PDB ID: 4zcf, chain B^39^), from *E. coli* bound to unmethylated DNA. Residues in the _184_NPPF_187_ motif that are critical for catalysis are labeled. (**C**) Comparison of the apo METTL16_291 (green sticks), SAH-bound METTL16_291 (yellow sticks) and SAH-bound METTL16_40–291 (PDB ID: 2h00^27^, blue sticks) structures revealed different backbone conformations of the _184_NPPFF_188_ motif; residue F188 could not be modeled in the METTL16_291/SAH structure. Side chains of P185 and P186 of METTL16_291/SAH are shown while the others have been removed for clarity. An arrow points to the *cis*-conformation between P185 and P186.

The general reaction mechanism catalyzed by m^6^A MTases has been established^37,38^. These enzymes contain a conserved [DNSH]PP[YFW] motif^38^, which corresponds to _184_NPPF_187_ in METTL16 (Figs. 4A and S1). Recent studies of METTL16_291 confirmed that conserved residues P185, P186 and F187 are essential for m^6^A methylation because METTL16_291 PP185/186AA and F187G mutants failed to methylate U6 and MAT2A RNA substrates *in vitro*^11^. Here, we propose a model of methyl transfer from SAM to the N6 amino group of adenosine by superposing the SAH-bound METTL16_40–291 structure with an m^6^A DNA MTase from *E. coli*, EcoP15l (PDB ID: 4zcf, chain B), in complex with an extrahelical adenosine acceptor (Fig. 4B)^39^. The protein chains of METTL16_40–291 and EcoP15l overlap with an rmsd value of 1.8 Å across 36 Cα atoms, and the acceptor 2′-deoxyadenosine appears poised for methyl transfer in the active site of METTL16_40–291/SAH modeled with SAM. Like other m^6^A MTases^21,37,38^, METTL16 likely uses the Oδ atom of N184 and carbonyl oxygen of P185 to negatively polarize the N6 amino group of adenosine via hydrogen bonding (Fig. 4A,B). As a result, the amino group becomes primed for transfer of the methyl group from SAM via an S_N_2 mechanism. The resulting *N*^6^-methylammonium adenosine cation could be stabilized by the same atoms that hydrogen bond to the amino group.

In general, m^6^A MTases use the aromatic residues Y, F or W in the [DNSH]PP[YFW] motif to hold the extrahelical, 180°-rotated nucleobase adenine in place by π-π stacking^37–39^. In our model, the methyl-acceptor adenosine is situated in a hydrophobic environment formed by L75 and F187, but F187 is not in an optimal conformation to stack with adenosine (Fig. 4B). However, with the proper substrates, METTL16 could likely reposition F187 for optimal π-π stacking interactions because _184_NPPFF_188_ undergoes a conformation rearrangement upon cofactor binding (Fig. 4C). Comparison of the apo METTL16_291, SAH-bound METTL16_291, and SAH-bound METTL16_40–291 structures revealed significantly different orientations of the _184_NPPFF_188_ motif. Notably, apo METTL16_291 and SAH-bound METTL16_40–291 show a *trans*-conformation between P185 and P186 whereas the SAH-bound METTL16_291 complex shows a *cis*-conformation (Fig. 4C). Additionally, the _184_NPPF_187_ residues are adjacent to a disordered region spanning 189–213 in the apo structure and 188–214 in the SAH-bound complex; these residues could not be modeled due to a lack of electron density. This disordered segment is one of the elements that distinguishes the Rossmann fold of METTL16 from other SAM-MTases (Fig. 1C,D). Hence, it may play an important role in RNA substrate recognition and/or orienting molecules for catalysis.

### METTL16 is a homodimeric RNA MTase

**Figure 5:**
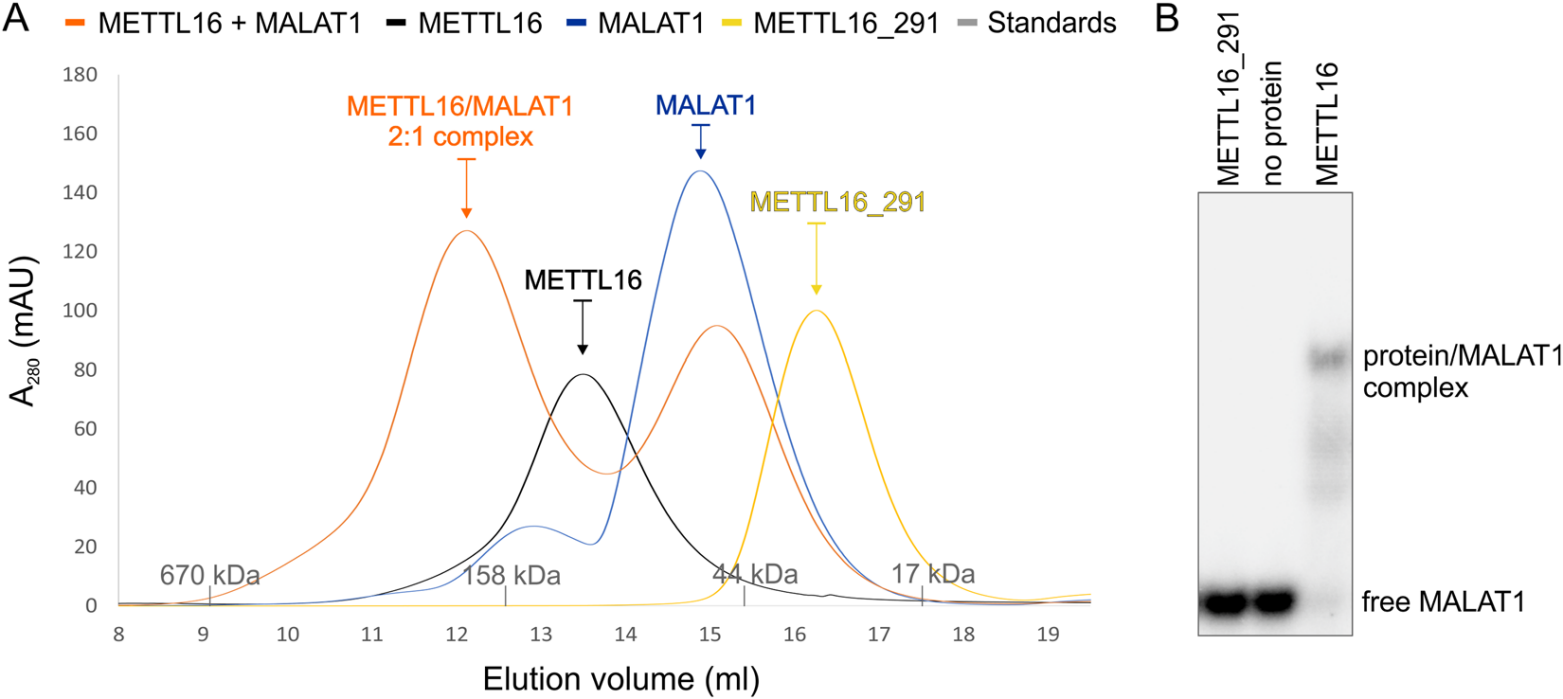
Analysis of METTL16-MALAT1 RNA triple helix interaction. (**A**) SEC revealed that METTL16 exists as a homodimer (SEC MW: ~103 kDa, black) while METTL16_291 is a monomer (SEC MW: ~31 kDa, yellow). SEC of the METTL16/MALAT1 RNA triple helix complex (SEC MW: ~188 kDa, orange) indicates a stoichiometry of 2:1 and also has a peak that elutes similar to the MALAT1 RNA triple helix (SEC MW: ~56 kDa, blue). Molecular weights of protein standards are shown as gray tick marks on *x*-axis. (**B**) Gel-shift assay showing that purified METTL16 (0.5 μM dimer) interacts with 5′-[^32^P]-labeled MALAT1 RNA triple helix (2 nM), whereas METTL16_291 (1 μM) does not. This gel image was cropped; the full-length gel image is shown in Fig. S3.

Most Class I SAM-MTases are monomeric, although some MTases form dimers or tetramers to recognize their substrates, to assemble their active sites or to facilitate catalysis^21,37^. For example, the heterodimeric METTL3/METTL14 complex forms a putative RNA-binding site at the METTL3-METTL14 interface^22–24^ and a METTL16 ortholog in *Caenorhabditis elegans* exists as a homodimer^40^. Therefore, we used size-exclusion chromatography (SEC) to examine the oligomerization states of both human METTL16 and METTL16_291. Our SEC data indicate that METTL16 exists as a homodimer because it elutes at a volume equivalent to a molecular weight of ~103 kDa, which is about twice the size of a single METTL16 polypeptide chain (theoretical MW 63.7 kDa) (Fig. 5A). Furthermore, its slower migration suggests that homodimeric METTL16 has a non-globular conformation. To further investigate the oligomerization of METTL16, we used SEC followed by small-angle X-ray scattering (SEC-SAXS). SEC-SAXS is a technique that uses X-ray scattering data of macromolecules in solution to determine their molecular weights, which is then used to deduce the oligomeric state of a complex eluting from the SEC column. Here, our SEC-SAXS analysis confirmed the homodimeric state of METTL16, determining a molecular weight of 125 kDa (theoretical MW 127.4 kDa) (Fig. S2A–C). In contrast, METTL16_291, whose theoretical molecular weight is 33.3 kDa, is a monomer, eluting from SEC at a volume equivalent to ~31 kDa (Fig. 5A). These results suggest that the C-terminal domain of human METTL16, which contains an evolutionarily variable region (residues 402–498) predicted to be disordered by MobiDB^41^, is required for oligomerization (Figs. 1B and S1). This finding is consistent with the oligomerization studies of the METTL16 ortholog in *C. elegans*^40^. Notably, homodimerization of METTL16 does not appear to be required for RNA binding and MTase activity because METTL16_291 methylates U6 and MAT2A RNA substrates *in vitro*^11^.

Recently, METTL16 was shown to interact with the RNA triple helix at the 3′ end of MALAT1^14^. Therefore, we used a native electrophoretic mobility shift assay (EMSA) to determine if METTL16 and METTL16_291 can interact with the MALAT1 RNA triple helix *in vitro*. Our results show that monomeric METTL16_291 (1 μM) does not bind to the MALAT1 RNA triple helix but METTL16 (0.5 µM dimer) does bind (Fig. 5B). Next, we used SEC and SEC-SAXS to examine the stoichiometry of the METTL16/MALAT1 RNA triple helix complex. SEC data showed that MALAT1 elutes at a volume equivalent to a molecular weight of ~56 kDa (Fig. 5A) whereas SEC-SAXS determined a molecular weight of 36 kDa (theoretical MW 30.2 kDa) (Fig. S2D–F). The apparent higher molecular weight of MALAT1 RNA is consistent with previous SEC studies showing that RNAs behave as globular proteins up to five fold larger than their theoretical molecular weights^42^. When METTL16 is mixed with the MALAT1 RNA triple helix at a 1:1 molar ratio, the SEC profile showed two peaks: one peak corresponding to the METTL16/MALAT1 RNA complex at ~188 kDa and one peak corresponding to unbound RNA at ~51 kDa (Fig. 5A). SEC-SAXS was used to examine the METTL16/MALAT1 RNA peak, and these results revealed a molecular weight of 155 kDa (theoretical MW 157 kDa), indicating that the METTL16 homodimer interacts with one molecule of MALAT1 RNA (Fig. S2G–I). Thus, these EMSA, SEC and SEC-SAXS results suggest that METTL16 may utilize different mechanisms to interact with different RNAs because only dimeric METTL16 interacts with the MALAT1 RNA triple helix but monomeric METTL16_291 binds and methylates both U6 and MAT2A RNAs^11^.

### Structural comparison of METTL16_291 and METTL3/METTL14

**Figure 6:**
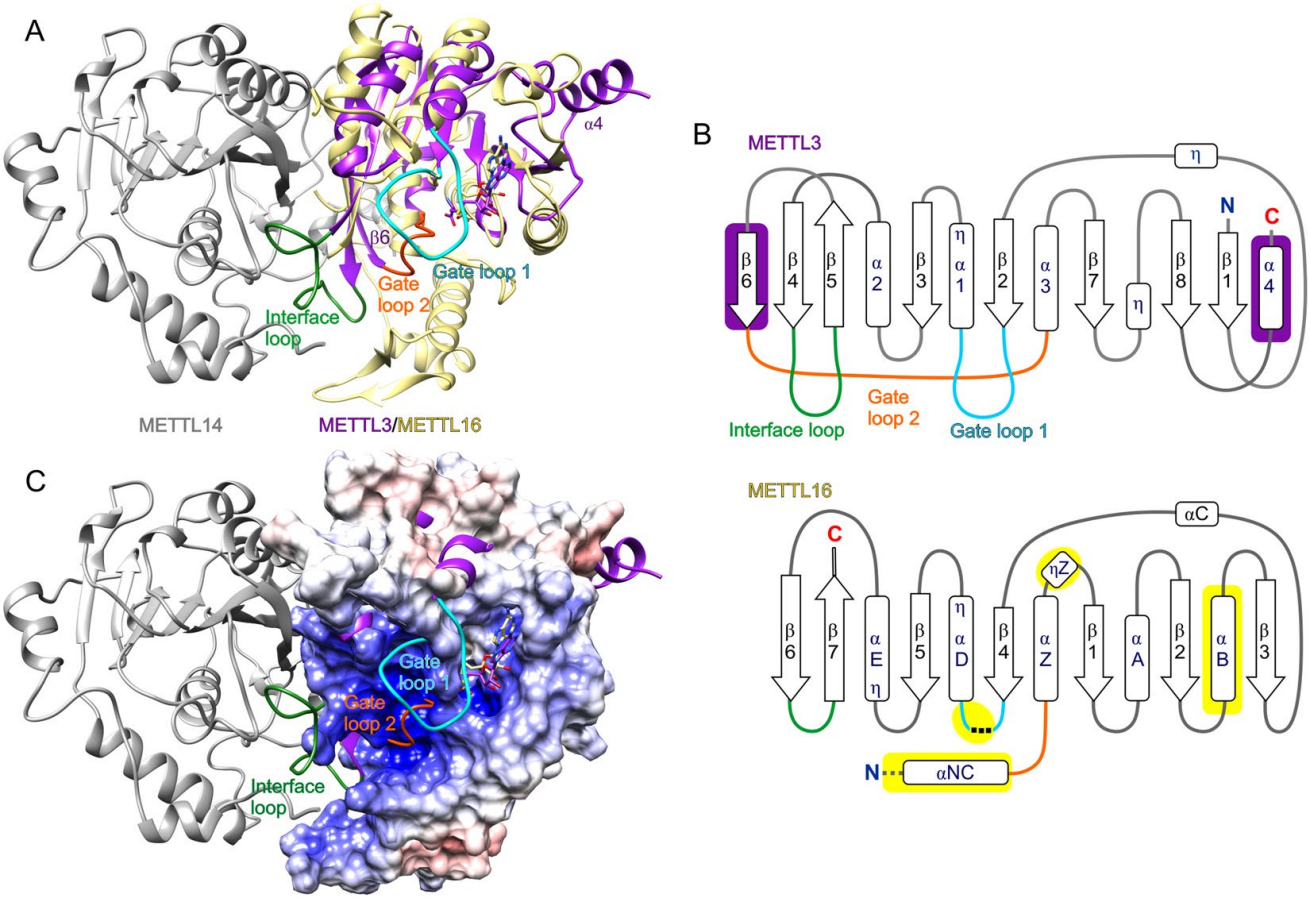
Structural comparison of METTL16_291 and METTL3/METTL14 complex. (**A**) METTL16_291/SAH (yellow cartoon) superposed on the human METTL3 (purple cartoon)/METTL14 (gray cartoon)/SAH complex (PDB ID: 5il2^22^). Gate loops and the interface loop of METTL3 are colored as follows: gate loop 1 is cyan, gate loop 2 is orange and interface loop is green. The unique β6 strand and α4 structures of METTL3 are labeled. (**B**) Schematic representation of Rossmann fold from METTL3 (residues 369–570) and METTL16 (residues 49–291). Arrows represent β-strands and boxes represent α and 3_10_ (η) helices. Elements unique to METTL3 are shown on purple background, elements unique to METTL16 are shown on yellow background and elements of METTL16 analogous to the gate loops and interface loop of METTL3 are colored as defined in panel A. Black dashed lines represent residues 188–214 that cannot be modeled and gray dashed lines represent N-terminal structural elements of METTL16 omitted for clarity. Helices of METTL3 are numbered as previously described^22^. (**C**) Surface electrostatic potential of METTL16_291/SAH is superposed on the two gate loops and interface loop of the METTL3/METTL14 (purple cartoon/gray cartoon) complex (PDB ID: 5il2^22^).

METTL16 is the second catalytically active m^6^A mRNA MTase to be validated in humans^11^. The only other m^6^A mRNA MTase currently known is the heterodimeric METTL3/METTL14 complex, whereby METTL3 has MTase activity and METTL14 functions as a scaffold to bind RNA^22–24^. Therefore, we superposed the X-ray crystal structures of human METTL3 (PDB ID: 5il2, residues 369–570) and human METTL14 (PDB ID: 5il2, residues 117–420) in complex with SAH^22^ onto SAH-bound METTL16_291. This comparative structural analysis revealed that METTL16_291 is similar to only METTL3 (rmsd 2.1 Å across 40 Cα atom pairs) and not METTL14 (Fig. 6). In METTL3, its conserved Rossmann fold includes four α helices, three 3_10_ helices and an eight-stranded parallel β sheet organized as ‘18723546’, with the β5 strand being antiparallel^22^ (Fig. 6A,B). This β-sheet organization is distinctly different from that of METTL16_291 (‘3214576’ in Fig. 6B), which explains the lack of sequence similarity between METTL16 and METTL3. However, the 3D spatial positioning of β strands and α helices within the Rossmann fold is similar except for the unique β6 strand and α4 helix of METTL3 and the unique αB helix of METTL16 (Fig. 6B). The SAH-binding sites in METTL3 and METTL16_291 are conserved in the Rossmann fold, although residue identities are different. Nonetheless, METTL16 and METTL3 form noncovalent interactions with the backbone atoms of homocysteine and N6, N1, 2′-hydroxyl and 3′-hydroxyl groups of adenosine in SAH.

The structural basis of RNA recognition by METTL16 and METTL3/METTL14 remains unclear. However, it has been proposed that the gate loops of METTL3/METTL14 play a role in RNA substrate recognition^22^. These gate loops map to the same face of the protein structure as the positively charged groove of METTL16_291 (Fig. 6C). More precisely, gate loop 1 of METTL3 (residues 395–410) corresponds to residues 184–222 in METTL16, a predicted loop that includes disordered residues 188–214 (Fig. 6B). These regions include the conserved catalytic residues: _395_DPPW_398_ in METTL3 and _184_NPPF_187_ in METTL16. The structural counterpart of gate loop 2 in METTL3 (residues 507–515) is the loop between αNC and αZ as well as N-terminal part of αZ in METTL16_291 (Figs. 1C,D and 6B). Both of these regions contribute to the positively charged groove (Fig. 6C). Interestingly, the long interface loop of METTL3 (residues 462–479) that interacts with METTL14 corresponds to the short β hairpin between strands β6 and β7, which contains R279 involved in the K47-R279 claw-like structure, in METTL16 (Figs. 3A and 6B). Thus, this comparative analysis shows that structural elements unique to METTL3 and METTL16 are located primarily in the RNA-binding site, providing a structural basis for why METTL3/METTL14 and METTL16 recognize different RNA substrates^6,7,10–12,22^.

### Role of METTL16 as an m^6^A RNA MTase

mRNA modifications, especially m^6^A, have been intensely studied in the past few years. Much of the work has focused on METTL3/METTL14, although it is now clear that METTL16 also plays a key role in m^6^A biology^11–13^. Recent studies suggest that METTL16 does not follow trends generally associated with m^6^A: (i) RNA targets do not have a RRACH motif and (ii) majority of METTL16-dependent m^6^A marks are found in introns^1,8,11,12,43^. These findings might not be that surprising considering METTL16 knockdown accounts for a ~20% loss of the m^6^A methylome in 293A-TOA cells^11^. Nonetheless, METTL16, which is distributed throughout the nucleus of HeLa cells, interacts with a wide range of RNAs, including long ncRNA, mRNA, miRNA, rRNA, snRNA and snoRNA^11,12,14^. Validated m^6^A targets include U6 snRNA, whose methyl mark at A43 is apparently essential for spliceosome activity, and MAT2A mRNA; both RNAs are methylated by METTL16 in a UACm^6^_AGA sequence^11–13^. METTL16 also interacts with the MALAT1 RNA triple helix, which is 13 base pairs from a weak m^6^A signal near A8290 in a ACAm^6^_ACA sequence^14,44^. Thus, it will be interesting to determine if the MALAT1 RNA triple helix itself or adjacent regions are substrates of METTL16 because MALAT1 is a cancer-promoting long ncRNA^45^. Furthermore, our studies show that full-length METTL16 is required to bind to the MALAT1 RNA triple helix, suggesting that the C-terminal domain expands the RNA interactome of METTL16. Two regions of the C-terminal domain of METTL16 (residues 289–399 and 514–562) are conserved in vertebrates^11^ while the N-terminal MTase domain is conserved from *E. coli* through human (Figs. 1B and S1). METTL16_291 shares 31% identity and 50% similarity to a METTL16 homolog in *E. coli*: RlmF (or *ybiN*), which methylates A1618 in 23 S rRNA^15^. Both METTL16 and RlmF can install m^6^A marks within the ACm^6^_AGR sequence^11,13,15^. Human METTL16 interacts with 18 S and 28 S rRNAs, which contain m^6^A marks at A1832 and A4220, respectively^12,14,46–48^. These marks occur within a GUAm^6^_ACR sequence, which would appear to be a suboptimal substrate for METTL3/METTL14 and maybe METTL16. Interestingly, the Am^6^_ACR motif found in rRNA also appears at A8290 in MALAT1 RNA. Another factor that may influence RNA substrate recognition and MTase activity is protein cofactors. Thus far, La protein, La-related protein 7, and methylphosphate capping enzyme have been identified as binding partners of METTL16 in a U6 snRNA-dependent manner^12^. Thus, more cellular, biochemical, and structural studies of METTL16 are necessary to elucidate its function at the molecular level.

## Conclusions

Our work expands the structural knowledge of human m^6^A RNA MTases by analyzing the ligand-binding sites and proposing a structural model of methyl transfer catalyzed by METTL16. Interestingly, METTL16 has an extensive, positively charged groove to bind RNA and this groove is largely comprised of structural elements unique to METTL16, suggesting this area may contribute to the RNA substrate specificity of METTL16 that is distinctly different from METTL3/METTL14. Moreover, our SEC and SEC-SAXS data show that METTL16 is a homodimer; however, the N-terminal domain of METTL16 is a monomer, indicating that the C-terminal domain facilitates protein dimerization. Homodimeric METTL16 is required to bind to the MALAT1 RNA triple helix while monomeric METTL16_291 is sufficient to methylate U6 and MAT2A RNAs^11^. Hopefully, future studies will reveal the biochemical and structural basis of the unique RNA substrate specificity of METTL16 and its roles as an m^6^A ‘writer’ and ‘reader’.

## Methods

### Cloning, overexpression and purification of METTL16_291 and METTL16

The sequences encoding human METTL16_291 (residues 1–291) and METTL16 (residues 1–562) [UniProt ID: Q86W50] were amplified by PCR using a pcDNA3-METTL16 plasmid and the following primers: Forward: 5′-TACTTCCAATCCAATGCCATGGCTCTGAGTAAATCAATGCATGCAA-3′ and Reverse: 5′-TTATCCACTTCCAATGTTACTAATCATAAAAACTCCAAGCTAAGGCC-3′ for METTL16_291; Forward: 5′-TACTTCCAATCCAATGCCATGGCTCTGAGTAAATCAATGCATGCAA-3′ and Reverse: 5′-TTATCCACTTCCAATGTTACTAGTTAACTGCAACAAGCCTGAAAATTTG-3′ for METTL16. Then, the DNA insert was incorporated into the pMCSG68 vector (Midwest Center for Structural Genomics) using a ligase-independent cloning method^49^. The expressed METTL16_291 and METTL16 proteins have an N-terminal His_6_-tag followed by a Tobacco Etch Virus (TEV) protease cleavage site and a Ser-Asn-Ala linker, which is encoded in the pMCSG68 vector. The sequence of the gene was confirmed by DNA sequencing (University of Notre Dame Genomics Facility).

Protein overexpression was carried out in BL21 Gold *E. coli* (Agilent Technologies) cells grown in 2 L LB media supplemented with 150 μg/ml ampicillin. The bacteria were cultured with shaking at 190 RPM at 37 °C until the OD_600_ exceeded 1.0 (~4 h). Afterwards, cultures were chilled to 18 °C, and protein expression was induced by the addition of 0.5 mM isopropyl-D-thiogalactopyranoside for 18 h. Cells were harvested by centrifugation at 5,000 x g for 15 min at 4 °C, resuspended in ice-cold binding buffer [50 mM HEPES-NaOH pH 7.5 at RT, 500 mM NaCl, 20 mM imidazole and 1 mM tris(2-carboxyethyl)phosphine (TCEP)] and stored at −80 °C.

Thawed cells were disrupted by sonication (4 min total of probe working time), using 4-s bursts with 26-s intervals for cooling in an ice/water bath. Lysate was cleared by centrifugation at 26,000 x g for 40 min at 4 °C. The supernatant was transferred to a 50-ml column packed with 3 ml of pre-washed HisTrap HP resin (GE Healthcare) and incubated for 5 min at 4 °C. The column was connected to a Vac-Man Laboratory Vacuum Manifold (Promega) to wash the protein-bound resin six times with 50 ml binding buffer for each wash. The His_6_-tagged protein was eluted by gravity with 20 ml elution buffer (50 mM HEPES-NaOH pH 7.5 at RT, 500 mM NaCl, 400 mM imidazole and 1 mM TCEP). The imidazole concentration was lowered to 20 mM and the His_6_-tag was cleaved with TEV protease (final concentration 0.1 mg/mL) during dialysis for 18 h at 4 °C. The sample was reapplied onto a HisTrap HP resin to remove both the cleaved His_6_-tag and the His_6_-tagged TEV protease. Flow-through was collected and concentrated to 2.0 ml using Amicon ultra centrifugal filters (30-kDa cutoff, Millipore). The protein was further purified by SEC (HiLoad 16/600 Superdex 200 pg, GE Healthcare) using size exclusion buffer (SEB) containing 25 mM HEPES pH 7.5 at RT, 100 mM KCl, 50 mM NaCl and 1 mM TCEP. The peak fractions of METTL16_291 and METTL16 were pooled and concentrated to 27 mg/ml and 10 mg/ml, respectively. A_280_ values were measured and molarity calculated using an extinction coefficient of 43890 and 63370 L/(mole·cm) for METTL16_291 and METTL16, respectively.

### Crystallization and diffraction data collection

Crystals grew at 19 °C using the hanging-drop method. The crystallization drop contained 4 µl METTL16_291 (27 mg/ml) and 2 µl of reservoir solution, which contained 1.3 M K_2_HPO_4_, 45 mM NaH_2_PO_4_, pH 8.5. Crystals measuring approximately 0.3 × 0.3 × 0.2 mm appeared after 3 days. To obtain the SAH-bound METTL16_291 complex, 0.2 µl of 200 mM SAM solution (Sigma-Aldrich, buffered in 50 mM HEPES pH 7.5) was added to the drop with mature crystals. Crystals were cryoprotected by soaking in crystallization solution supplemented with 25% ethylene glycol, vitrified in liquid nitrogen and stored until data collection. Diffraction data were collected under cryocooled conditions (100 K) at the Advanced Photon Source, Argonne National Laboratory, on beamline 22-ID. XDS^50^ package was used for data reduction. The statistics of the data collection and processing are summarized in Table 1.

### Determination and refinement of the crystal structures

The crystal structures of METTL16_291 were solved by molecular replacement using Phaser^51^ and the X-ray crystal structure of human methyltransferase 10 domain containing protein (94% identity, PDB ID: 2h00^27^). ARP/wARP^52^ was used to build the initial model, which afterwards was placed inside the unit cell with the ACHESYM server^53^. Manual fitting in the electron density maps was completed in COOT^54^ with iterative rounds of model refinement in Refmac^55^. Ten and four TLS^56,57^ groups were added for apo and SAH-bound METTL16_291, respectively, as recommended by the TLSMD server^58^. The refinement statistics are listed in Table 1. Our final models include residues 3–291 (missing 189–213) in apo METTL16_291 and 5–291 (missing 188–214) for SAH-bound METTL16_291. The presence of SAH, rather than SAM, was determined based on the electron density maps calculated for refined models with either of the two ligands. No peak of positive density appeared at the position corresponding to the methyl group when SAH was placed in the model, whereas for SAM, the methyl group lied in a peak of negative density. A sodium cation in the apo structure was modeled on the basis of octahedral coordination sphere (with four out of six electron pair donors) and the interatomic distances that refined to values below 2.4 Å. Three and four ethylene glycol molecules were modeled in the apo and SAH-bound METTL16_291 structures, respectively.

### Analytical SEC

The 94-nt MALAT1 RNA triple helix 5′-GGAAGGUUUUUCUUUUCCUGAGAAAACAACACGUAUUGUUUUCUCAGGUUUUGCUUUUUGGCCUUUUUCUAGCUUAAAAAAAAAAAAAGCAAAA-3′ was generated using the pHDV-MALAT1 ENE + A WT plasmid as described previously^14,59^. All samples (METTL16_291, METTL16, MALAT1 RNA triple helix and METTL16/MALAT1 RNA triple helix) were diluted to ~21 µM in 100 µl total volume of SEB buffer supplemented with 1 mM MgCl_2_. RNA was denaturated at 95 °C for 1 min, snap-cooled on ice for 5 min and incubated at room temperature for 1 h. METTL16 and the MALAT1 RNA triple helix were mixed at a 1:1 ratio of 21 μM:21 μM and incubated at room temperature for 45 min prior to injection. Samples were injected onto a Superdex 200 10/300 GL column (GE Healthcare) pre-equilibrated with SEB buffer supplemented with 1 mM MgCl_2_ and resolved at a flow rate of 0.6 ml/min. Thyroglobulin (670 kDa), gamma-globulin (158 kDa), ovalbumin (44 kDa) and myoglobin (17 kDa)) were used as standards (Gel Filtration Standard, BioRad). Molecular weights of METTL16_291, METTL16, MALAT1 RNA triple helix and METTL16/MALAT1 RNA triple helix complex were calculated using a standard curve.

### Small-angle X-ray scattering (SAXS) measurements

Solution SAXS data were collected at the Advanced Photon Source BioCat, 18-ID beamline in the SEC-SAXS pipeline^60^. Samples pre-equilibrated in SEB buffer supplemented with 1 mM MgCl_2_ were injected onto the SEC column at the following concentrations: 30 μM METTL16, 25 μM MALAT1 RNA and METTL16 + MALAT1 RNA complex at a 2:1 ratio of 30 μM:15 μM. Data were recorded at room temperature on a PILATUS 3 1 M detector with 0.5-s exposures every 3 s at 1.0 Å wavelength. Sample cell-to-detector distance was 3.5 m, and the data range was 0.0163 Å^−1^ to 0.3666 Å^−1^ (q = 4π sin θ/λ, where 2θ is the scattering angle, and λ is the X-ray wavelength). Baseline scattering was averaged from data points measured before and after the protein peak, whereas sample frames corresponding to the elution peak were averaged to maximize the signal-to-noise ratio. Data were processed using the BioCAT beamline pipeline, which is based on *ATSAS* package^61^. Guinier analysis was performed in *PRIMUS*^62^ and *GNOM*^63^ for calculation of the radius of gyration (*Rg*) and the pair distribution function, *P(r)*. Molecular masses are based on the calculation of low-resolution *ab initio* models with the use of *DAMMIF*^64^, *DAMAVER*^65^, *DAMMIN*^66^, and *DAMFILT*.

### Electrophoretic mobility shift assay (EMSA)

The MALAT1 RNA triple helix was 5′-end radiolabeled with [γ-^32^P]ATP as described previously^14^. Immediately before incubating RNA with protein, RNA was denaturated at 95 °C for 1 min, snap-cooled on ice for 5 min, and incubated at room temperature for 1 h. Then, 2 nM 5′-[^32^P]-labeled MALAT1 RNA was mixed with 1 μM METTL16_291 or METTL16 in binding buffer (25 mM Tris pH 7.5, 25 mM NaCl, 150 mM KCl, 1 mM MgCl_2_, 1 mM DTT, 0.5 mg/ml tRNA, 1U/μl RNase Inhibitor and 8% glycerol) at room temperature for 45 min. The reactions were resolved using 5–6% native polyacrylamide gel (19:1 acrylamide:bisacrylamide, 1 × Tris-Borate buffer (TB), 1 mM MgCl_2_) and running buffer (0.75 × TB, 1 mM MgCl_2_). Electrophoresis was run under an electric field of 8 V cm^−1^ for 2.5 h. Gels were visualized on phosphor screen and scanned using an Amersham Typhoon IP Biomolecular Imager (GE Healthcare).

### Other software used

Disordered region of METTL16 was calculated by MobiDB server^41^. Molecular illustrations were created using UCSF Chimera^67^, which also calculated rmsd values across pairs of Cα atoms within 3 Å radius for all superposition analyses. Surface electrostatic potential was calculated using the PDB2PQR server^33^. The amino acid sequences of METTL16 from 394 chordates were aligned using ClustalX2^68^ and conservation calculated using the ConSurf server^69^.

### Accession numbers

Coordinates and structure factors were deposited in the Protein Data Bank (PDB): apo METTL16_291, PDB ID: 6b91; METTL16_291/SAH complex, PDB ID: 6b92.

## Electronic supplementary material

Supplementary Information
