# Supplementary material for: Structural insights into the RNA methyltransferase domain of METTL16
Source: Sci Rep. 2018 Mar 28;8:5311. doi: 10.1038/s41598-018-23608-8 (PMC5871880; doi:10.1038/s41598-018-23608-8)
Supplement: Supplementary file 1 — Supplementary Information [file 41598_2018_23608_MOESM1_ESM.pdf]

SUPPLEMENTARY INFORMATION

Structural insights into the RNA methyltransferase domain of METTL16

Agnieszka Ruszkowska, Milosz Ruszkowski, Zbigniew Dauter and Jessica A. Brown

Supplementary Figures

Figure S1. Sequence conservation of METTL16 among chordates.

Figure S2. Small-angle X-ray scattering measurements of METTL16/MALAT1 RNA complex.

Figure S3. Uncropped gel image of native gel-shift assay.

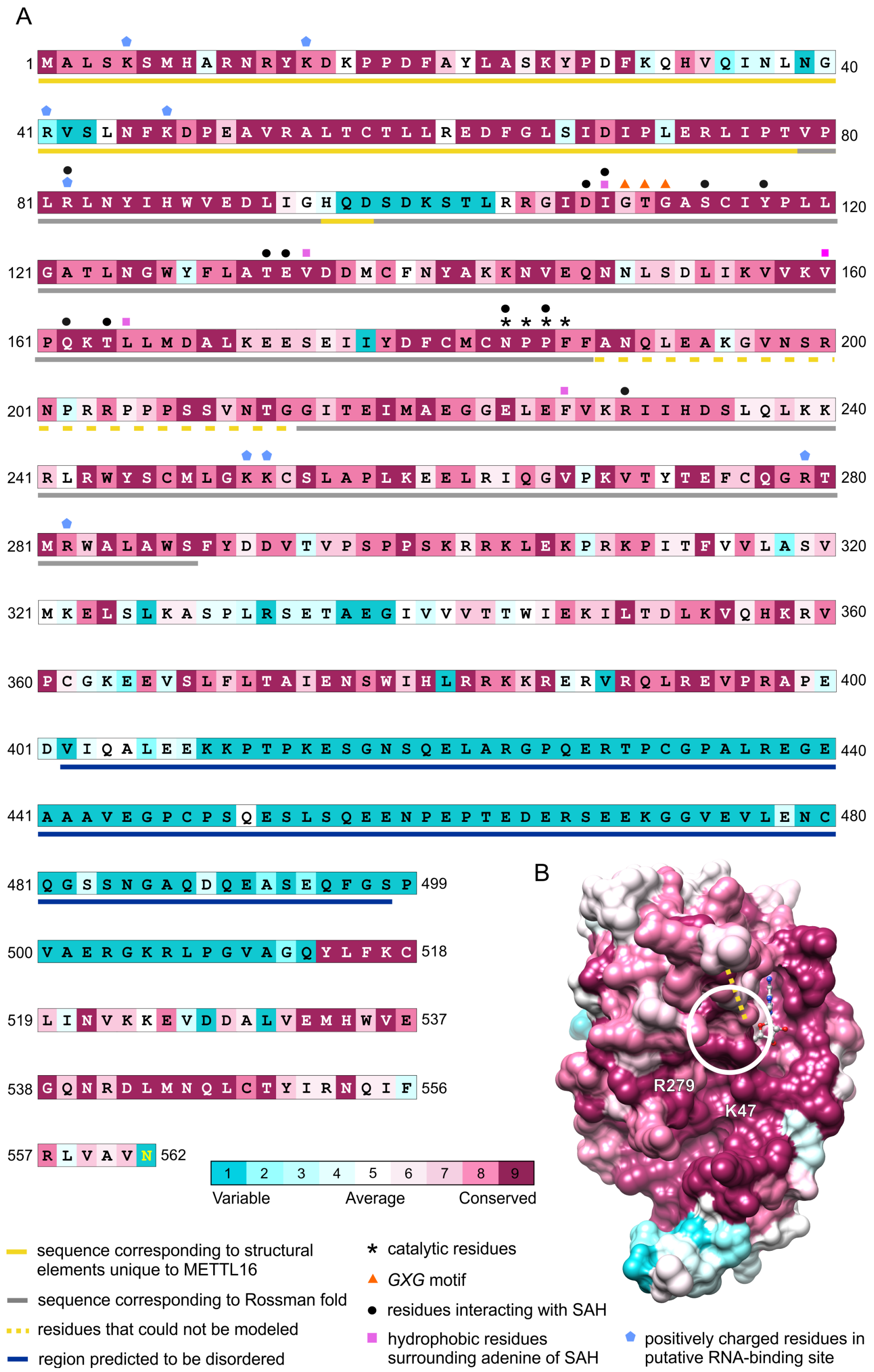

Figure S1. Sequence conservation of METTL16 among chordates. The amino acid sequences of METTL16 from 394 chordates were aligned using ClustalX2[68] and conservation calculated using the ConSurf server[69]. The conservation heat map was applied to both A) the primary structure of human METTL16 (yellow N562 indicates insufficient data to calculate conservation score) and B) the surface representation of the METTL16_291/SAH complex. A conservation score of 9 (purple) represents ~98-100% similarity. White circle indicates location of catalytic residues $_{184}$NPPF$_{187}$. The yellow dashed lines mark residues that could not be modeled. All other symbols are defined in the legend.

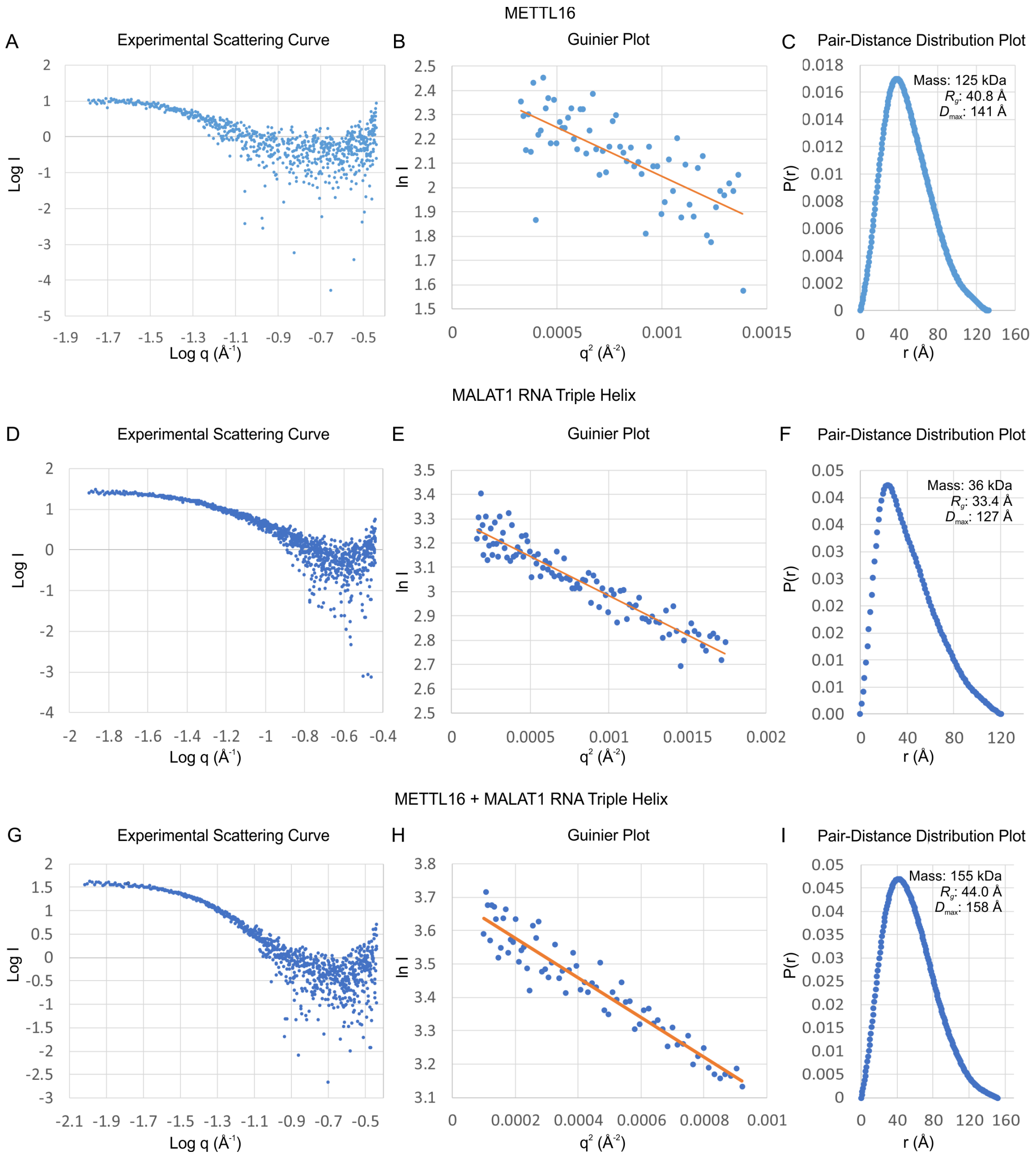

Figure S2. Small-angle X-ray scattering measurements of METTL16/MALAT1 RNA complex. Three samples were evaluated: METTL16 (A-C), MALAT1 RNA triple helix (D-F), and METTL16 in complex with MALAT1 RNA triple helix (G-I). The experimental scattering data (A, D, G) are plotted as the log of scattering intensity (I) versus the log of momentum transfer (q); $q = 4\pi\sin(\theta)/\lambda$, where 2θ is the scattering angle and λ is the X-ray wavelength. Guinier plots (B, E, H) display the natural log of I as a function of q^2. The linear fit satisfies qR_g of ≤ 1.3, where R_g is the radius of gyration. Panels C, F and I show the pair-distance distribution functions (P(r)) of intraparticle distances (r), with the calculated molecular masses, radii of gyration (R_g) and maximum intraparticle distances (D_{max}) displayed in the upper-right corner.

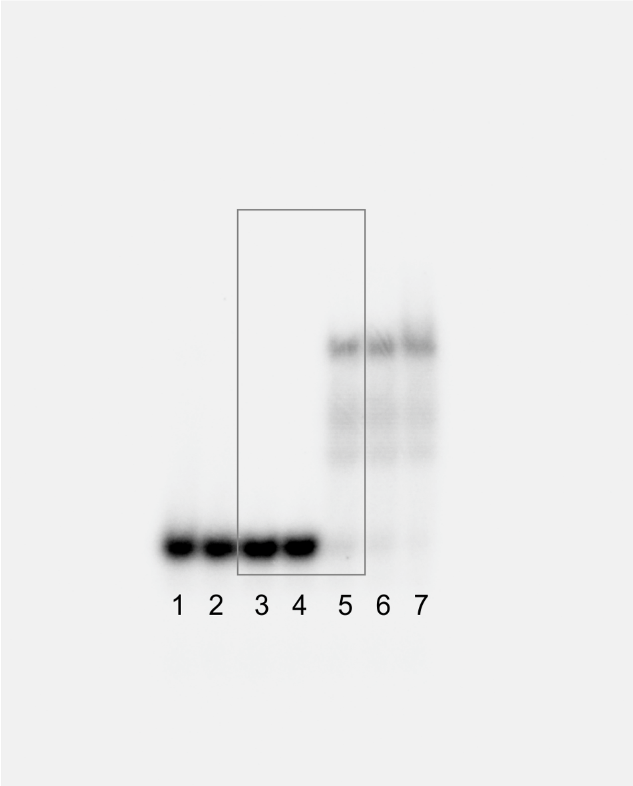

Figure S3. Uncropped gel image of native gel-shift assay. The full-length gel image is shown for the native gel-shift assay in Figure 5B. The box demarks cropped region. All lanes contain 2 nM 5′-[^{32}P]-labeled MALAT1 RNA triple helix and the following proteins:
1) 4 μM METTL16_291
2) 2 μM METTL16_291
3) 1 μM METTL16_291
4) no protein
5) 0.5 μM METTL16
6) 1 μM METTL16
7) 2 μM METTL16
Please note, the concentration of dimeric METTL16 is shown. Also, up to 4 μM of METTL16_291 does not interact with the MALAT1 RNA triple helix.
